# Anti-bacterial and anti-viral nanchangmycin displays anti-myeloma activity by targeting Otub1 and c-Maf

**DOI:** 10.1038/s41419-020-03017-4

**Published:** 2020-09-30

**Authors:** Yujia Xu, Tong Sun, Kun Zeng, Min Xu, Jinhao Chen, Xiaofeng Xu, Zubin Zhang, Biyin Cao, Xiaowen Tang, Depei Wu, Yan Kong, Yuanying Zeng, Xinliang Mao

**Affiliations:** 1 Guangdong Institute of Cardiovascular Diseases, Guangdong Key Laboratory of Vascular Diseases, the Second Affiliated Hospital; Guangdong Key Laboratory of Protein Modification and Degradation, School of Basic Medical Sciences, Guangzhou Medical University, Guangzhou, 511436 P. R. China; 2 Department of Pharmacology, College of Pharmaceutical Sciences, Soochow University, Suzhou, Jiangsu 215123 P. R. China; 3 Department of Neurology, the First Affiliated Hospital of Soochow University, Suzhou, Jiangsu 215100 P. R. China; 4 Department of Hematology, Zhangjiagang Hospital of Soochow University, Zhangjiagang, 215620 China; 5 Department of Hematology, the First Affiliated Hospital of Soochow University, Suzhou, Jiangsu 215100 P. R. China; 6 Department of Urology, Nanjing Jinling Hospital, School of Medicine, Nanjing University, Nanjing, Jiangsu 210002 P. R. China; 7 Department of Oncology, Suzhou Municipal Hospital, Suzhou, Jiangsu 215100 P. R. China

**Keywords:** Pharmacology, Targeted therapies, Myeloma

## Abstract

As a deubiqutinase Otub1 stabilizes and promotes the oncogenic activity of the transcription factor c-Maf in multiple myeloma (MM), a malignancy of plasma cells. In the screen for bioactive inhibitors of the Otub1/c-Maf axis for MM treatment, nanchangmycin (Nam), a polyketide antibiotic, was identified to suppress c-Maf activity in the presence of Otub1. By suppressing Otub1, Nam induces c-Maf polyubiquitination and subsequent degradation in proteasomes but does not alter its mRNA level. Consistently, Nam downregulates the expression of CCND2, ARK5, and ITGB7, the downstream genes regulated by c-Maf, and promotes MM cell apoptosis as evidenced by PARP and Caspase-3 cleavage, as well as Annexin V staining. In line with the hypothesis, overexpression of Otub1 partly rescues Nam-induced MM cell apoptosis, and interestingly, when Otub1 is knocked down, Nam-decreased MM cell survival is also partly ablated, suggesting Otub1 is essential for Nam anti-MM activity. Nam also displays potent anti-MM activity synergistically with Doxorubicin or lenalidomide. In the in vivo assays, Nam almost completely suppresses the growth of MM xenografts in nude mice at low dosages but it shows no toxicity. Given its safety and efficacy, Nam has a potential for MM treatment by targeting the Otub1/c-Maf axis.

## Introduction

Multiple myeloma (MM) is a hematological malignancy derived from plasma cells^1^. In the past two decades, various anti-MM drugs have been successfully developed and marketed, including inhibitors of histone deacetylases^2^, immunomodulators^3^, monoclonal antibodies^4^, proteasomal inhibitors^5^, and selective inhibitors of nuclear export^6^. These drugs alone or in combination have benefited hundreds and thousands of MM patients. However, the relapsed and refractory cases are frequently reported. Because MM is highly heterogenetic^7^, development of novel therapeutics by taking advantage of the progress in MM pathophysiology is of interest.

The Maf family proteins including c-Maf, MafB, and MafA are a class of basic leucine zipper transcription factors, of which c-Maf is highly expressed and promotes MM survival and predicts poor outcomes of MM patients^8^. c-Maf overexpression in MM is associated with the t(14;16) chromosomal translocation^9^, and other events including the IL6-STAT3^10^ and the FGFR3 signaling pathways^11^. Our recent studies showed that the stability of c-Maf protein is mainly regulated by the ubiquitin-proteasome pathway^12^. c-Maf undergoes degradation in proteasomes after the K48-linked polyubiquitination mediated by the ubiquitin ligase HERC4^13^, the ubiquitin-conjugating enzyme UBE2O^14^ or the TMEPAI/NEDD4 ligase^15^. Moreover, c-Maf ubiquitination is a dynamic process, deubiquitinases USP5 and USP7 could prevent c-Maf from ubiquitination therefore stabilizing c-Maf and promoting MM cell survival and proliferation^16,17^. The ubiquitin thioesterase Otub1 is recently found to stabilize c-Maf by preventing its K48-linked polyubiquitination^18^. In the present study, we reported that nanchangmycin (Nam), an antibiotic for the treatment of Gram-negative bacteria^19^ and Zika virus infection^20^, could inhibit the Otub1/c-Maf axis, therefore promoting c-Maf degradation and inducing MM cell apoptosis at both in vitro and in vivo.

## Results

### Identification of Nam as an inhibitor of the Otub1/c-Maf axis

**Fig. 1:**
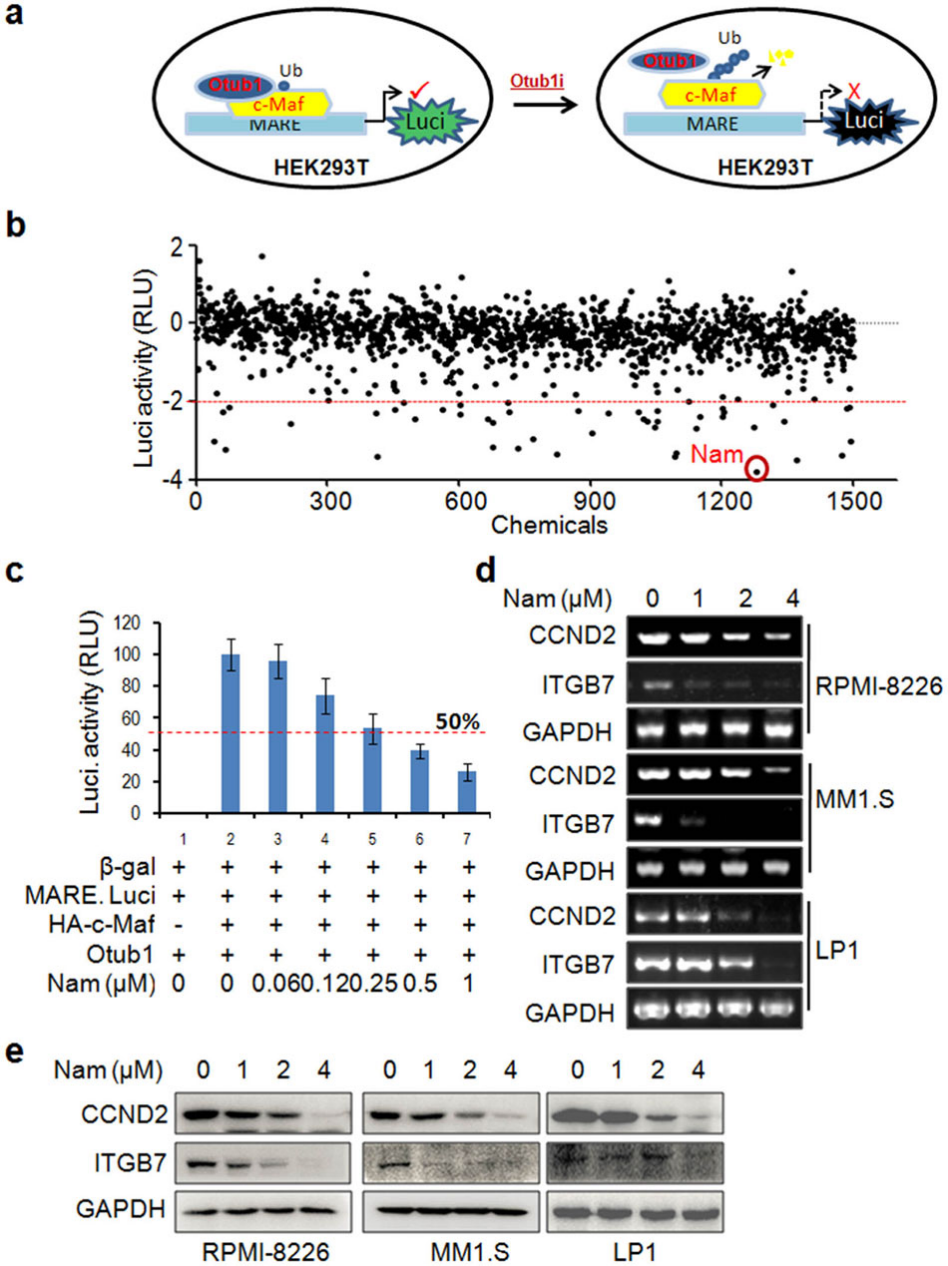
Identification of Nam as an inhibitor of the c-Maf transcriptional activity in the presence of Otub1. **a** The system for screening inhibitors of the c-Maf/Otub1 axis. **b** HEK293T cells expressing c-Maf, Otub1, and MARE.Luci were seeded in 96-well plates overnight and treated with 5 µM of each compound from the natural product library for 24 h before being lysed for luciferase assays. The activities of the compounds were expressed as log2 (sample RLU/control RLU). Drugs associated with a value of log2 < −2 were considered potential inhibitors. **c** HEK293T cells co-transfected with the HA-c-Maf, pMARE.Luci with Otub1 plasmids were treated with Nam for 24 h followed by luciferase activity measurements. **d**, **e** MM cell lines were treated with Nam for 24 h, followed by measuring the expression levels of CCND2 and ITGB7 by both RT-PCR (**d**) and IB (**e**) assays. GAPDH was used as the internal control. All experiments were repeated three times.

We recently found that Otub1 prevents K48-linked ubiquitin conjugates from c-Maf as its deubiquitinase and inhibition of Otub1 leads to c-Maf degradation and MM cell apoptosis^18^, suggesting the Otub1/c-Maf axis is a potential target for MM treatment. To identify inhibitors of the Otub1/c-Maf axis, HEK293T cells expressing c-Maf, Otub1 and Maf-recognition element-driven luciferase (MARE.Luci) (Fig. 1a) were incubated with 5 µM of each compound from the TargetMol® Natural Product Library. The results showed that some agents including doxorubicin, homoharringtonine, ouabain, sanguinarine hydrochloride, and Nam were able to strikingly suppress the activity of luciferase (Fig. 1b), suggesting these agents might inhibit c-Maf transcriptional activity in the presence of Otub1. Because doxorubicin and homoharringtonine have been widely used for the treatment of MM and other hematological malignancies, while ouabain and sanguinarine are reported to induce leukemia or MM cell death^21,22^, Nam that remains largely unknown thus was chosen in the present study. To confirm this finding, HEK293T cells transfected with plasmids of c-Maf, Otub1, MARE.Luci as well as beta-gal (as an internal control) were treated with Nam at various concentrations for 24 h. The luciferase analyses revealed that Nam inhibited the activity of MARE-driven luciferase in a concentration-dependent manner and the IC_50_ was 0.265 ± 0.07 µM (Fig. 1c). To find out whether Nam suppressed c-Maf activity in MM cells, three typical MM cell lines RPMI-8226, MM1.S, and LP1 were treated with Nam from 1 to 4 µM for 24 h, followed by IB and RT-PCR assays. The results showed that Nam downregulated the expression of c-Maf targeted genes CCND2 and ITGB7 at both mRNA (Fig. 1d) and protein levels (Fig. 1e). All these results thus concluded that Nam inhibits c-Maf transcriptional activity.

### Nam induces c-Maf polyubiquitination and degradation in the proteasomes

**Fig. 2:**
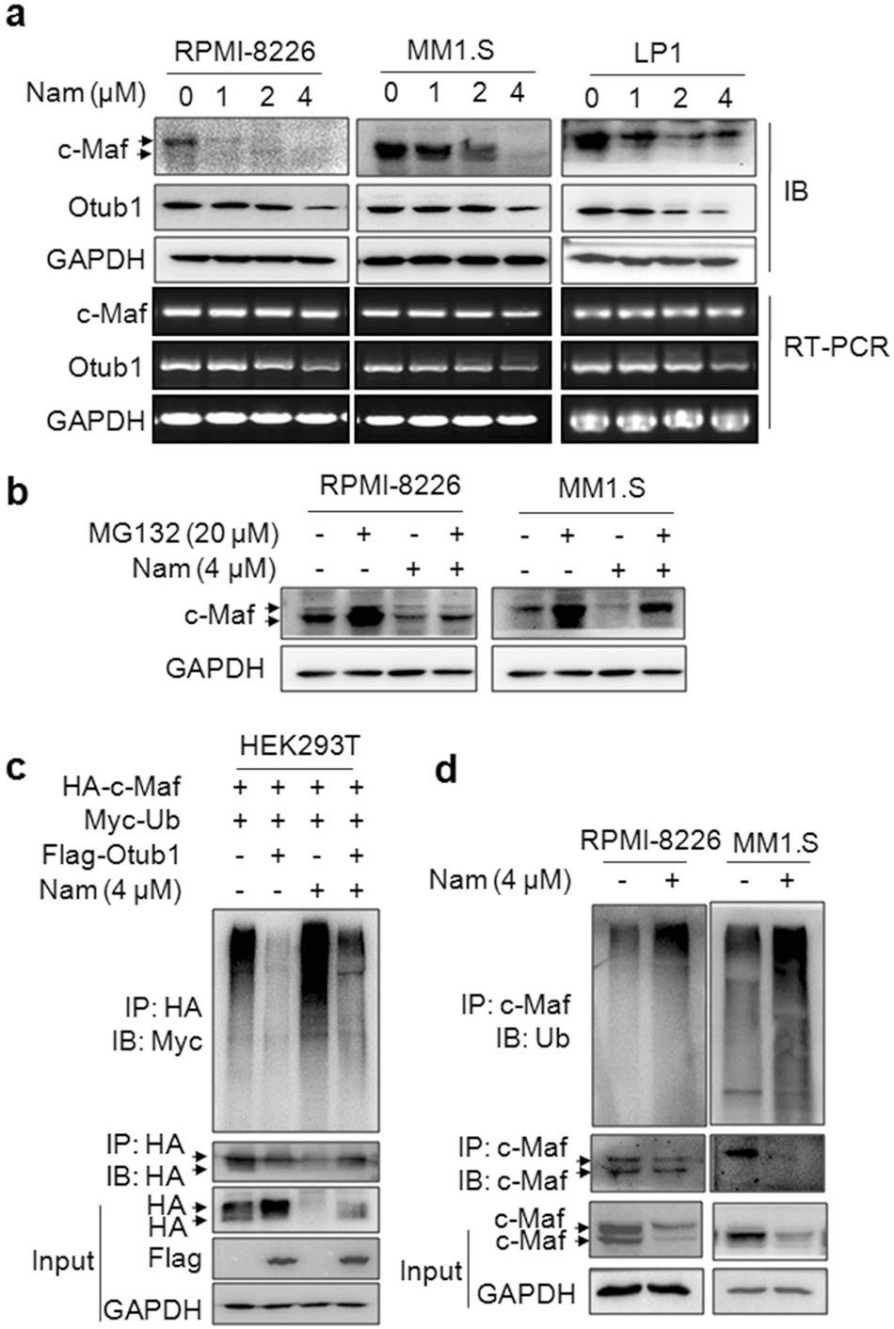
Nam induces c-Maf polyubiquitination and degradation in the proteasomes. **a** RPMI-8226, MM1.S and LP1 cells were treated with Nam at indicated concentrations for 24 h, followed by IB and RT-PCR assays for c-Maf and Otub1. **b** MM1.S and RPMI-8226 cells were treated with Nam with or without MG132 for 24 h, followed by IB assays. **c** HEK293T cells were co-transfected with the HA-c-Maf, Flag-Otub1, and Myc-Ub plasmids for 48 h. Cells were then treated with Nam for 24 h before being lysed for IP/IB assays. **d** MM1.S and RPMI-8226 cells were treated with Nam for 24 h, followed by IP/IB assays.

Because Nam was identified from the screen in the presence of Otub1, we next wondered whether Nam could interfere with c-Maf ubiquitination and degradation. To this end, MM cells were treated with Nam followed by IB and RT-PCR assays against c-Maf protein and mRNA, respectively. The results showed that Nam markedly downregulated the expression of c-Maf protein but not its mRNA (Fig. 2a), suggesting Nam promotes c-Maf protein degradation and has no effects on its transcription. Next, we wondered whether Nam-induced c-Maf degradation was via the ubiquitin-proteasome pathway. To this end, MM cells were treated with Nam, MG132 alone or together for 24 h. The following IB assays showed that Nam significantly decreased c-Maf protein but it was markedly rescued by MG132 (Fig. 2b), suggesting that Nam mediated c-Maf degradation in the proteasomes. Moreover, Otub1 is a deubiquitinase of c-Maf, we thus wondered whether Nam could abolish the effects of Otub1 on c-Maf ubiquitination. To this end, HEK293T cells transfected with c-Maf, Ub, and Otub1 were treated with Nam for 24 h followed by IP/IB assays. As shown in Fig. 2c, Otub1 prevented c-Maf from polyubiquitination, while Nam increased c-Maf ubiquitination and partly rescued the ubiquitination level in the presence of Otub1 (Fig. 2c), suggesting that Nam antagonized Otub1 in terms of c-Maf polyubiquitination. Lastly, we evaluated the effects of Nam on c-Maf ubiquitination in MM cells. As shown in Fig. 2d, Nam strikingly increased the polyubiquitination levels of c-Maf in both RPMI-8226 and MM1.S cells. These results thus collectively showed that Nam promotes c-Maf polyubiquitination and degradation in the proteasomes in association with inhibition of Otub1.

### Nam decreases MM cell viability and induces MM cell apoptosis

**Fig. 3:**
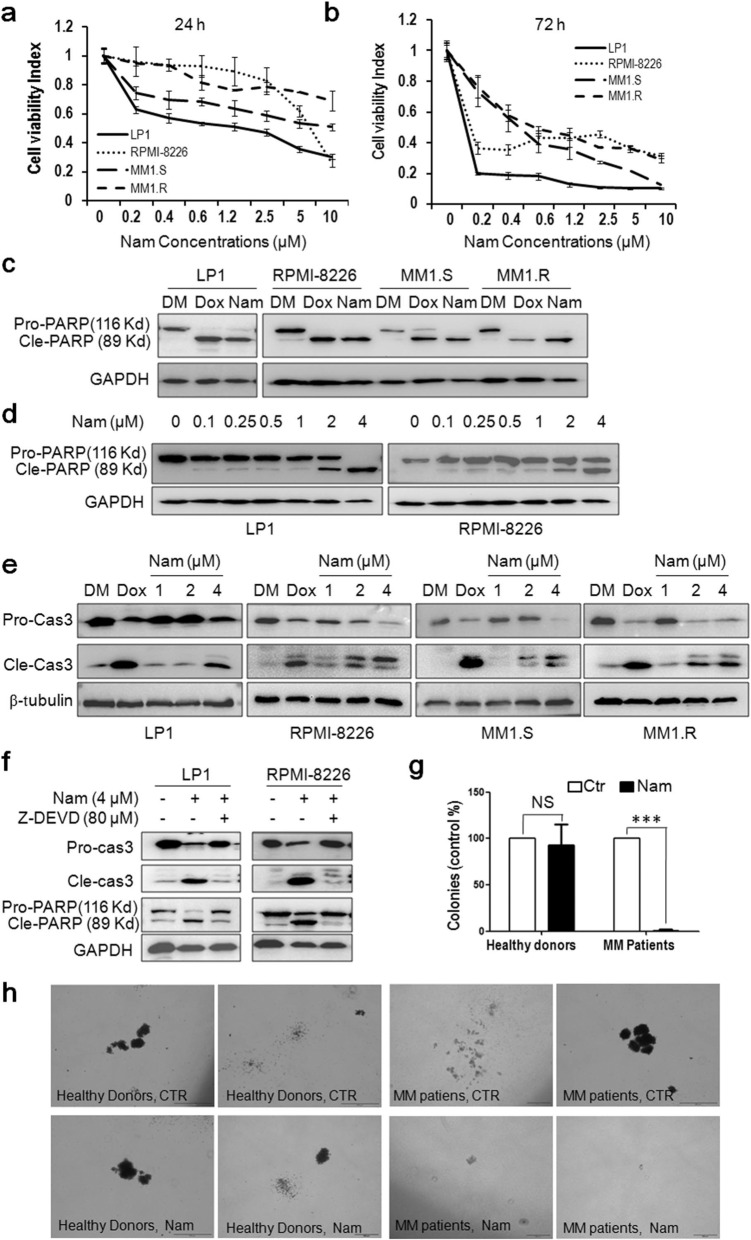
Nam decreases MM cell viability and induces their apoptosis. **a**, **b** MM cells were incubated with Nam for 24 h (**a**) and 72 h (**b**) followed by MTT assay. **c** MM cell lines were treated with DMSO (DM), Nam (4 µM) or doxorubicin (Dox, 1 µM) for 24 h followed by IB analyses. **d** LP1 and RPMI-8226 cells were treated with Nam for 24 h, followed by IB assay. **e** MM cells were treated with Dox (1 µM) or Nam for 24 h, followed by Caspase-3 (Cas-3) cleavage analysis. **f** LP1 and RPMI-8226 cells were treated with Nam and Z-DEVD for 24 h, followed by IB assay against Caspase-3 and PARP. **g** Bone marrow mononuclear cells from MM patients (*n* = 20) or healthy donors (*n* = 5) were mixed with Nam-containing MethoCult^TM^ medium, followed by seeding in 3.5-cm plates. Colonies containing >20 cells were counted on day 14 from 20 microscopic fields. **h** Representative colonies from (**g**) were photographed. ***p < 0.001. GAPDH and β-tubulin were used as internal loading controls.

Because inhibition of c-Maf leads to MM cell apoptosis^16^, while Nam downregulates c-Maf, we next examined the effects of Nam on MM cell viability. MM cell lines LP1, RPMI-8226, MM1.S, and MM1.R were treated with Nam for 24 and 72 h. The following MTT assay revealed that Nam reduced cell viability within 24 h (Fig. 3a) and it became striking in 72 h in which the IC_50_ values for all cell lines examined were <1.0 µM (Fig. 3b). To find out whether Nam induced MM cell apoptosis, MM cell lines treated with Nam were subjected to IB assays for PARP cleavage, a hallmark of apoptosis. As shown in Fig. 3c, Nam induced marked cleavage of PARP in a manner similar to or higher than Dox, the positive control and a major anti-MM agent, suggesting Nam-induced MM cell apoptosis. Moreover, Nam could induce PARP cleavage at a concentration as low as 0.1 μM (Fig. 3d). In line with this finding, Nam induced the activation of Caspase-3, one of the key executive apoptotic enzymes. As shown in Fig. 3e, the cleaved forms (both 17 and 19 KD) of Caspase-3 were markedly increased, along with the decrease in the pro-Caspase-3 form, in a manner similar to Dox. To validate whether Nam-induced apoptosis was caspase-dependent, RPMI-8226 cells were co-treated with Nam and Z-DEVD, a specific inhibitor of Caspase-3, -7, -8, and -10. The subsequent IB analysis revealed that Z-DEVD almost completely abolished the cleavage of PARP and Caspase-3 (Fig. 3f), suggesting that Nam induced caspase-dependent apoptosis. We also evaluated the effects of Nam on colony formation of primary MM blood marrow stem cells. The CFU results showed that Nam significantly suppressed the colony formation of blood progenitor/stem cells from MM patients at 1 µM, but had no effects on the counterparts from the bone marrow of healthy donors (Fig. 3g, h).

**Fig. 4:**
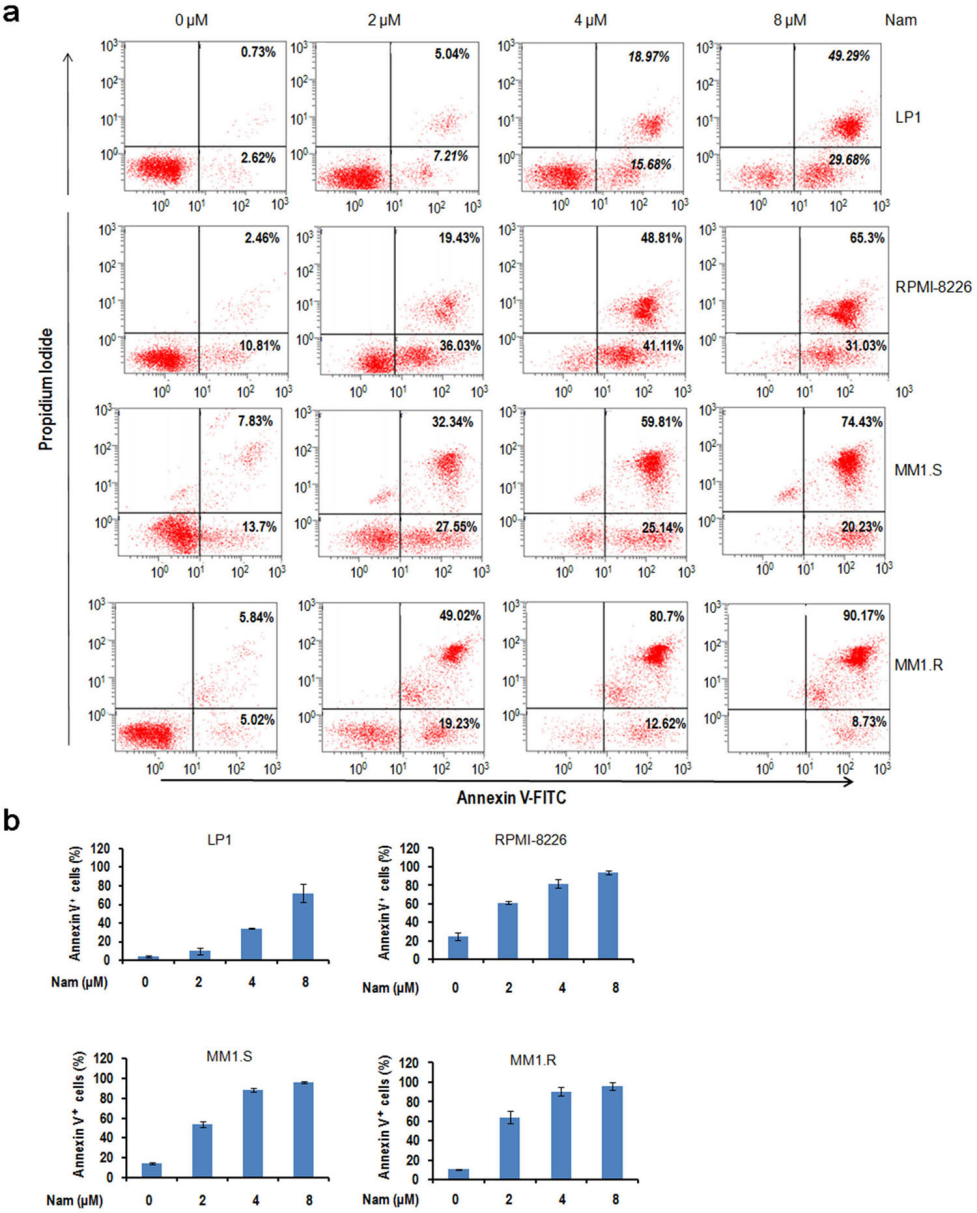
Nam induces MM cell apoptosis analyzed by flow cytometry. **a** LP1, RPMI-8226, MM1.S, and MM1.R cells were treated with Nam at indicated concentrations for 24 h. Cells were then stained with Annexin V-FITC and propidium iodide and analyzed on a flow cytometer. **b** The average percentages of Annexin V positive cells were expressed based on three independent flow cytometric analyses.

We also performed a flow cytometric analysis to measure the surface exposure of Annexin V, the gold standard of apoptosis. As shown in Fig. 4a, the Annexin V positive fractions were induced by Nam in a concentration-dependent manner, and the Annexin V positive fractions at 2 and 4 µM were drastically increased (Fig. 4b). Therefore, all these results collectively indicated that Nam displays potent activity in inducing MM cell apoptosis.

### Otub1 is important for Nam-induced MM cell apoptosis

**Fig. 5:**
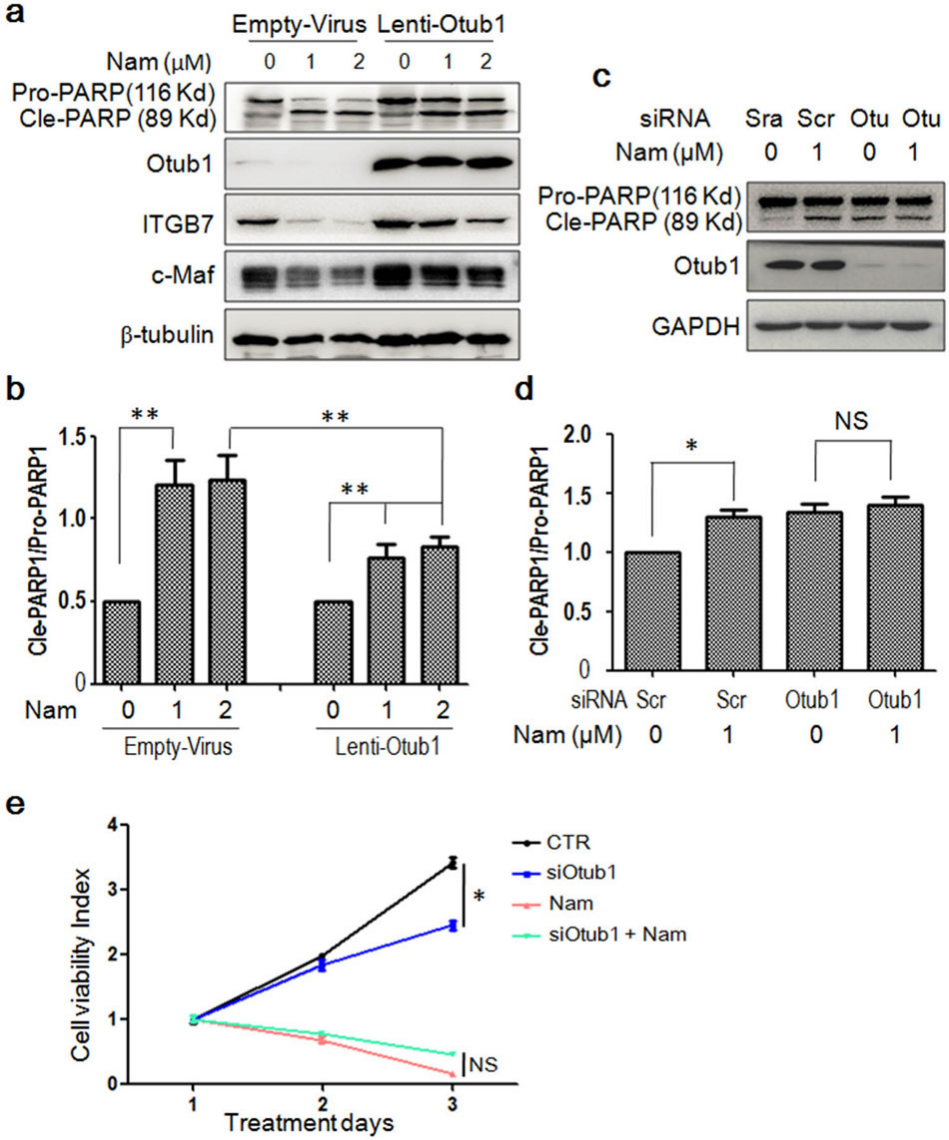
Otub1 is important for Nam-induced MM cell apoptosis. **a** RPMI-8226 cells were infected with empty lentivirus or lentiviral Otub1. After 72 h, cells were treated with Nam in a dose-dependent manner for another 24 h. Cells were then harvested for IB assays. **b** The ratio of cleaved PARP to pro-PARP band densities from (**a**). **c** RPMI-8226 was transfected with siOtub1 (Otu) for 48 h, followed by Nam or DMSO treatment for another 24 h. Cell lysates were subjected to IB assays. **d** The ratio of cleaved PARP to pro-PARP from (**c**). **e** RPMI-8226 cells were transfected with siOtub1 for 48 h, followed by treatment with Nam for 24 h, cell viability was measured by MTT assay. *p < 0.05. **p < 0.01. NS, not significant. All experiments were repeated for three times independently.

The above investigation suggests that Nam suppresses c-Maf oncogenic activity and induces MM cell apoptosis via inhibiting Otub1. To confirm this hypothesis, RPMI-8226 cells were infected with lentiviral Otub1 followed by IB assays for PARP and c-Maf-modulated proteins. As shown in Fig. 5a, Nam significantly decreased c-Maf protein, along with ITGB7, one of the representative genes regulated by c-Maf. However, infection of lentiviral Otub1 markedly increased c-Maf protein level and rescued the expression of ITGB7 (Fig. 5a). Accordingly, the anti-MM activity of Nam was partly blocked. As shown in Fig. 5a, b, PARP cleavage was markedly decreased by overexpressed Otub1. These results suggested that Otub1 is potentially critical for Nam to induce MM cell apoptosis. To verify this hypothesis, we next knocked down Otub1 from RPMI-8226 cells followed by Nam treatment. As shown in Fig. 5c, d, both Nam and Otub1 knockdown alone induced PARP cleavage, while in the absence of Otub1, PARP cleavage was partly decreased. This finding was also consistent with the viability assay. As shown the MTT assays in Fig. 5e, siOtub1 and Nam alone decreased MM cell viability, but cell viability induced by Nam was slightly increased when Otub1 was knocked down. All these findings thus collectively suggested that Otub1 is essential for MM cell survival and Nam-induced MM cell apoptosis.

### Small-dose Nam enhances MM cell apoptosis induced by lenalidomide and doxorubicin

**Fig. 6:**
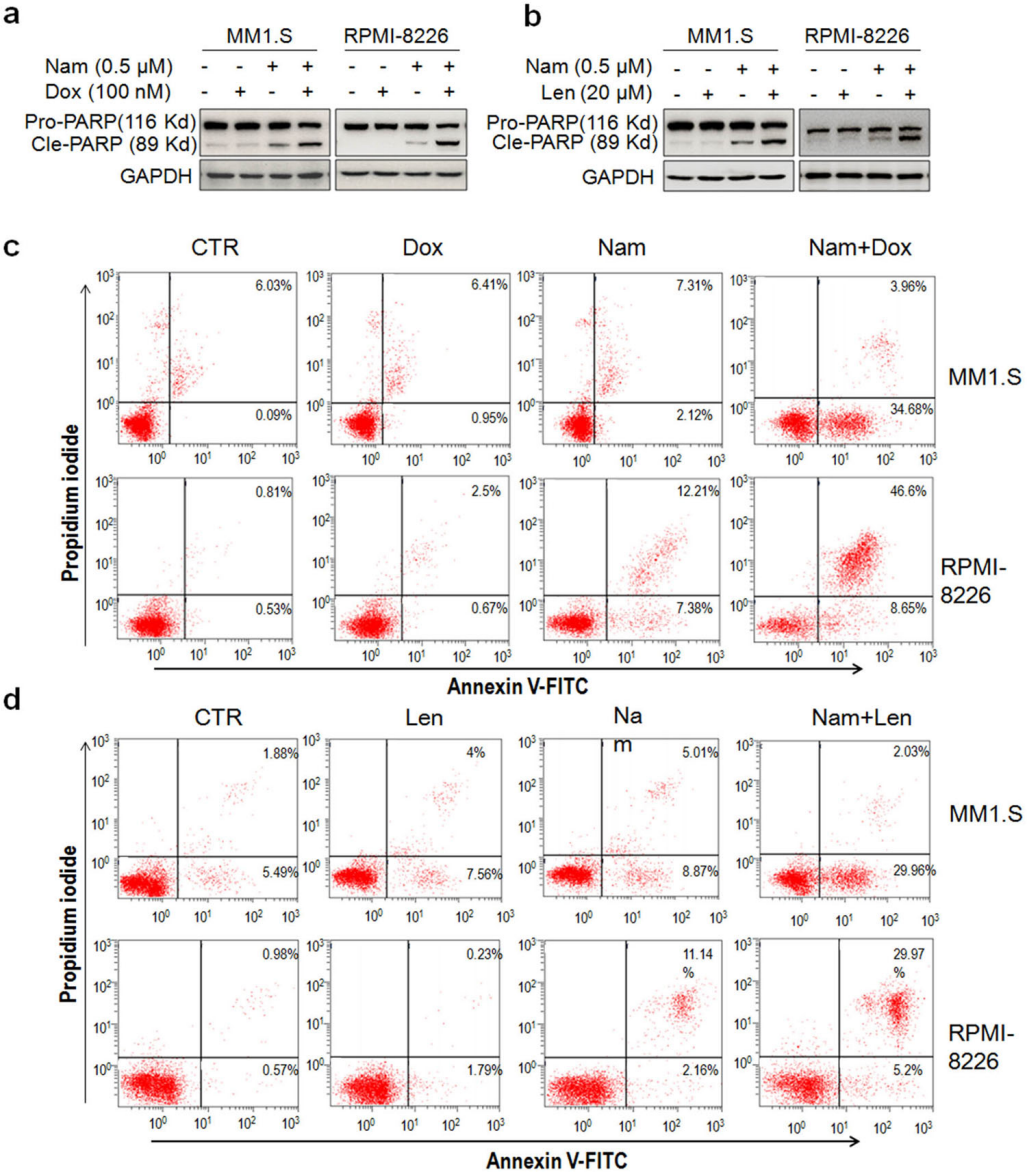
Nam synergizes with doxorubicin and lenalidomide to induce MM cell apoptosis. **a**, **b** MM1.S and RPMI-8226 cells were treated with Nam, doxorubicin (Dox) or lenalidomide (Len), alone or together, for 24 h, followed by IB assays to view the cleavage levels of PARP. **c** MM cells were treated with Nam and/or Dox for 24 h, followed by Annexin V-FITC and propidium iodide staining and flow cytometric analysis. **d** MM cells were treated with Nam and/or Len for 24 h, followed by Annexin V-FITC and propidium iodide staining and flow cytometric analysis. All experiments were repeated three times.

Because the combined therapy is a preferential choice to overcome potential resistance, we next examined whether Nam synergized with Dox and lenalidomide (Len), two major anti-MM drugs. MM cells were treated with low doses of Nam and Dox or Len. At the tested concentrations, neither Nam, Dox or Len could induce marked PARP cleavage, but the cleavage was significantly increased by the combined treatment of Nam with Dox or Len (Fig. 6a, b), suggesting that Nam synergized Dox or Len to induce MM cell apoptosis. To confirm this hypothesis, MM cells treated with the above drugs were subjected to Annexin V-FITC/PI staining and flow cytometric analyses. The results showed that neither Nam, Dox nor Len at low concentrations alone strikingly increased the fractions of Annexin V positive cells, but these fractions were significantly increased by combined treatments in both MM1.S and RPMI-8226 cell lines (Fig. 6c, d). This finding is consistent with the IB assays in terms of PARP cleavage. Therefore, Nam at low doses could synergize with clinical drugs for MM treatment.

### Nam impairs tumor growth in nude mice without overt toxicity

**Fig. 7:**
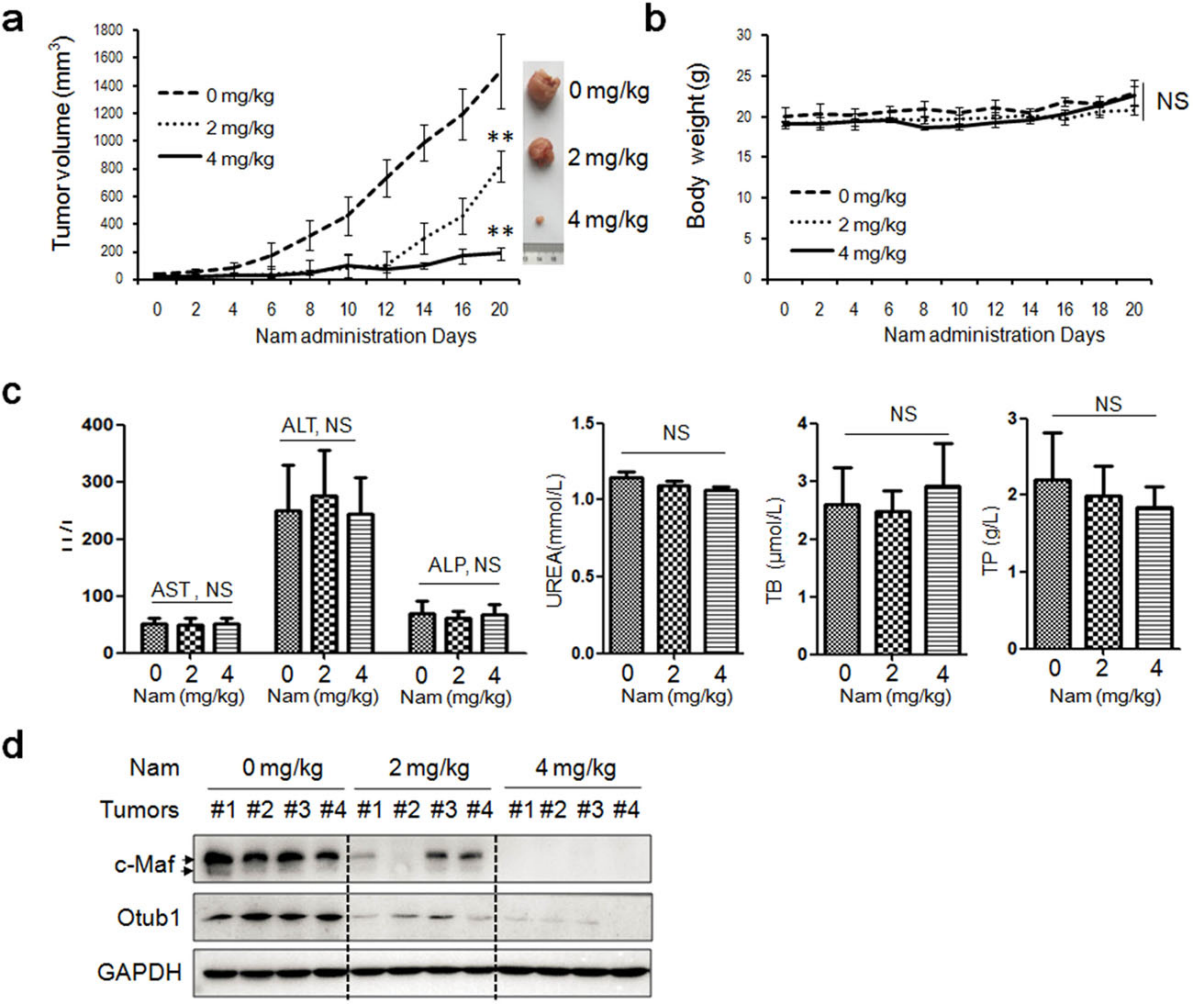
Nam impairs tumor growth in nude mice. **a** RPMI-8226 cells were subcutaneously inoculated into the right flank of female nude mice to establish a multiple myeloma xenografted model. When tumors were palpable, mice (*n* = 5 for each group) were orally administered vehicle or Nam (2 mg/kg or 4 mg/kg body weight) in CMC-Na for a continuous 20 d. During the experiments, tumor sizes were monitored every other day. **b** The body weight curve over the experiment. **c** The blood species from mice were subjected to measurement of the contents of AST, ALT, ALP, urea, total bilirubin (TB) and total proteins (TP). **d** Tumor tissues from xenografted mice were subjected to the measurement of c-Maf and Otub1 proteins by IB assays. **p < 0.001 and NS, not significant, compared with the control.

Lastly, we evaluated the anti-MM activity of Nam in vivo. RPMI-8226 cells were subcutaneously injected into the flanks of nude mice. When the tumors were palpable, mice were randomly divided into three groups (*n* = 5 for each group), each was orally received vehicle (CMC-Na), 2 or 4 mg/kg of Nam for continuous 20 d on a no-blinding base. As showed in Fig. 7a, tumors grew rapidly in the vehicle group, but the growth was strikingly suppressed by Nam and it was almost completely inhibited by Nam at the dosage of 4 mg/kg (Fig. 7a), in contrast, Nam had no effects on mice growth in terms of body weight (Fig. 7b) or biochemical assays including aspartate aminotransferase (AST), or alanine aminotransferase (ALT), alkaline phosphatase (ALP), urea, total bilirubin and total proteins (Fig. 7c), further demonstrating Nam was safe at least at the dosages examined. To find out the association of c-Maf and tumor decreases, we performed IB assays for c-Maf and Otub1 in tumor tissues. As shown in Fig. 7d, Nam markedly decreased c-Maf at both 2 and 4 mg/kg dosages. Out of our expectation, Nam also downregulated Otub1 in tumor tissues excised from nude mice, suggesting that Nam might also affect Otub1 expression in vivo. Therefore, Nam impairs MM growth in vivo in association with c-Maf degradation but without marked toxicity.

## Discussion

c-Maf has long been regarded as a therapeutic target of MM due to the following events: (1) c-Maf is highly expressed in MM promoted by chromosome translocation^9^, STAT3 and FGFR3 signaling transductions^10,11^, and others; (2) c-Maf transgenic mice develop myeloma-like syndrome^23^; (3) Genetic inhibition of c-Maf leads to MM cell death^16,24^; (4) c-Maf is mainly expressed in the early stage of embryonic development but not expressed in adult tissues except CD4 T cells^25^. However, as a transcription factor, c-Maf is hard to be targeted for MM treatment. Recent studies demonstrated that c-Maf could be turned over via the ubiquitin-proteasomal pathway^12^. Induction of c-Maf degradation is an efficient manner to induce MM cell death^16,26^. The OTU-domain family deubiquitinase Otub1 is recently demonstrated as a novel c-Maf deubiquitinase. Inhibition of Otub1 results in c-Maf degradation and MM cell apoptosis^18^. Based on these findings, the present study designed a drug screen based on the Otub1/c-Maf-driven luciferase and found that the antibiotic Nam can act as an inhibitor of the Otub1/c-Maf axis and it displays potent anti-MM activity in vitro and in vivo.

Nam is a broad spectrum polyether antibiotic produced by Streptomyces nanchangensis NS3226 and shows high activity against Gram-positive bacteria, West Nile, dengue, and chikungunya viruses, as well as malaria, nematodes, and some insects^20,27^. Currently, there are no reports on Nam in mammalian cells. The present study for the first time demonstrated that Nam is potent to decrease human MM cell viability and to induce MM cell apoptosis. In addition, Nam displays activities to induce MM cell apoptosis independent of their genetic alterations. Four representative MM cell lines were examined in the present study, including LP1 carrying t(4;14), RPMI-8226 carrying c-Myc insertion on t(16;22)(q32;q11):der(16) and t(14;16)^24,28^, MM1.S that is sensitive to dexamethasone, and MM1.R that lacks glucocorticoid receptor and is resistant to dexamethasone^29^. All these cells were sensitive to Nam and the common feature is that all these cell lines expressing c-Maf due to chromosomal translocations or other signaling dysregulations^24,30^. Nam also synergizes with Dox or Len, two major anti-MM drugs, at a very low concentration to induce MM cell apoptosis, suggesting its broad application potentials. Nam impairs the colony-forming of bone marrow cells from MM patients but not those from healthy donors. Moreover, Nam shows great anti-MM activity in vivo without overt toxicity. The blood biochemical analyses reveal that Nam has no marked changes on the liver functions in terms of typical biomarkers, such as AST, ALP, and ALT. This could be explained by that fact that Nam is used to promote the growth of poultry and ruminants^19,31^.

Nam displays various mechanisms in its anti-bacteria or anti-virus activities^20,32^. To kill bacteria, Nam adheres to bacteria and destroys their cell wall by oxidization, while Nam prevents Zika virus entry across cell membranes therefore inhibiting the virus infection^20^. In the present study, we found that Nam can suppress the Otub1/c-Maf axis. Because Otub1 is a deubiquitinase of c-Maf and inhibition of Otub1 leads to c-Maf degradation and MM cell death. The mechanistic investigation showed that Nam leads to c-Maf turnover via the ubiquitin-proteasomal pathway because when cells are treated with MG132, one of the most common proteasomal inhibitors, Nam-induced c-Maf degradation is abolished. Therefore, by rescuing c-Maf polyubiquitination and degradation, Nam suppresses the c-Maf transcriptional activity and induces MM cell apoptosis.

In conclusion, the present study for the first time demonstrated that Nam bears anti-cancer activity in addition to the known activity against virus, bacteria and parasites. Given its activity and safety, Nam could be developed for MM treatment alone or in combination with in-use drugs.

## Materials and methods

### Cell culture

HEK293T cells were cultured in DMEM (Hyclone®). RPMI-8226 from Procell (Wuhan, China) and LP1, MM1.S and MM1.R maintained in the lab were cultured in IMDM (Hyclone®), all were added with 10% FBS (ExCell Bio, Inc., Shanghai, China) and 1% penicillin/streptomycin (Sigma-Aldrich®). Primary bone marrow cells were donated by healthy donors and MM patients from the First Affiliated Hospital of Soochow University with written consent for research purposes and approved by the Review Board and Ethic Committee of Soochow University.

### Plasmids and siRNA transfection

The complete cDNA fragments for Otub1 and c-Maf were cloned into pcDNA3.1 using Phanta Max Super-Fidelity DNA polymerase (Vazyme Biotech Co., Ltd, Nanjing, China). The pMARE.Luci plasmid was constructed previously^15^. The siOtub1 (5′-GCAAGUUCUUCGAGCACUU-3′) was provided by Guangzhou RiboBio Co., Ltd (Guangzhou, China) transfected into MM cells by using Lipo2000 (Thermo Fisher®) according to the manufacturer’s instructions.

### Chemicals and antibodies

The natural product library was obtained from Target Molecule Corp., Wellesley Hills, MA. Nam was provided by MedChemExpress LLC, Princetion, NJ. Doxorubicin, lenalidomide, and MG132 were purchased from Sigma-Aldrich Chemicals Co. The antibodies included: anti-HA (M180-3), anti-Myc (M192-3), and anti-Flag (M185-3L) that were from MBL Biotech Co., Ltd. (Beijing, China). Anti-Otub1 (Ab175200) was from Abcam Shanghai, China. Anti-GAPDH (60004-1-Ig), anti-ITGB7 (18309-1-AP), and anti-c-Maf (55013-1-AP) were from Proteintech Group, Inc. (Wuhan, China). Anti-c-Maf (sc-7866) and anti-Ub (sc-271289) were from Santa Cruz Biotechnology, Inc. (Santa Cruz, CA, USA). Anti-PARP (#9532), anti-CCND2 (#3741), and anti-Caspase-3 (#9662) were from Cell Signaling Technology, Danvers, MA. Anti-β-tubulin (AC008) was obtained from ABclonal Biotechnology Co., Ltd, Woburn, MA.

### Screening of the natural chemical library

HEK293T cells stably co-transfected with the c-Maf, Otub1, and pMARE.Luci plasmids were seeded into 96-well plates (1 × 10^4^/well). After overnight growth, cells were treated with each compound (5 µM) from the Natural Compound Library (Target Molecule Corp) for 24 h. The cells were then collected for the luciferase activity assay by using Bright-Glo^®^ system (Promega, Madison, WI, USA) as described previously^13^. The activities of the compounds were expressed as log2 (sample RLU/control RLU). Compounds associated with a value of log2 < −2 were considered potential inhibitors and were taken for further analyses.

### Immunoblotting (IB) analysis

Total proteins were extracted by using a 0.5% SDS-containing protein lysis buffer. Protein concentrations were determined by the BCA assay. Equal amounts of protein (30 µg) were separated by SDS-PAGE and transferred to polyvinylidene difluoride membranes. The blots were probed with appropriate antibodies, as described previously^15^.

### Immunoprecipitation (IP)

After being treated with Nam for 24 h, cells were harvested to prepare whole cell lysates, and the IP assay was conducted as described previously^17^. After IP, proteins were re-suspended in RIPA buffer and used for an IB assay.

### Cell viability assay and flow cytometry

MM cells were treated with Nam for 24–72 h before being subjected to the MTT assay as described previously^17^. Apoptosis was measured by flow cytometry to determine Annexin V and propidium iodide (MultiSciences Biotech Co., Ltd, Hangzhou, China) staining as described previously^17^.

### Reverse transcription-polymerase chain reaction (RT-PCR)

The RT-PCR was performed as described previously^15^. The primers for Otub1 were 5′-TGTTTCTATCGGGCTTTCG-3′ (forward) and 5′-AGATGTGCGGATTGGTGG-3′ (reverse). The specific primers for c-Maf, GAPDH, CCND2, and ITGB7 were described previously^15^. PCR products were visualized by GoldView® staining (TransGen Biotech Co., Ltd., Beijing, China) following electrophoresis on 2% agarose gels.

### Otub1 lentivirus construction

The Otub1 cDNA generated by using the following primers: 5′-CCGCTCGAGATGGCGGCGGAGGAACCTCAG-3′ (forward) and 5′-CGCGGATCCTCTTTGTAGAGGATATCG-3′ (reverse) was inserted into pLVX-AcGFP lentiviral vector (Clontech Laboratories) within the *Xho*I and *BamH*I sites. To generate lentiviral particles, HEK293T cells at 80% confluence were transfected with pLVX-AcGFP-Otub1 (10 µg) as well as plasmids for VSV-G envelope glycoprotein (3.5 µg), Rev (2.5 µg), and ΔR8.74 packaging proteins (6.5 µg) using Lipofectamine2000 (Invitrogen) as described as previously^17^. Forty hours later, the lentiviral particle-enriched supernatants were harvested, filtered and stored frozen at −80 °C for further use. The pLVX-AcGFP lentiviral particle was used as a mock control in the infection assay.

### Colony-forming assay

The colony-forming unit assay was performed as described previously^33^. Briefly, bone marrow cells (6.25 × 10^5^/mL) were mixed with Nam-containing MethoCultGF H4434 medium (StemCell Technologies, Vancouver, Canada) followed by being seeded in 3.5-cm plates and were cultured for 14 d. Colonies containing >20 cells were counted for statistical analysis. Colony images were also photographed.

### Myeloma xenografts in nude mice

Female BALB/c nude mice (5–6 weeks old) purchased from Shanghai Slac Laboratory Animal Co. Ltd (Shanghai, China) were used to establish MM xenograft models with the human MM cell line RPMI-8226. When tumors were palpable, mice were randomly divided into three groups, each was administrated vehicle, Nam (2 or 4 mg/kg body weight), respectively, on a daily base for 20 d. Body weight and tumor volumes were measured every other day (tumor volume = tumor length × width^2^/2). At the end of this experiment, tumors and blood samples were collected for further investigation. All experiments involving mice were reviewed and approved by the Review Board of Animal Care and Use of Soochow University.

### Biochemistry analyses of blood samples

At the end of the experiment, whole blood samples were collected from the eyes and were immediately subjected to evaluate the serum levels of physiochemical indexes including ALT, AST, ALP, urea, total bilirubin, and total proteins. All biochemical assays were performed using a clinical automatic chemistry analyzer.

### Statistical analysis

All experiments were independently performed in three times except the xenograft study in which each model containing 5 mice (*n* = 5). Statistical difference between the control and the experimental groups was analyzed by student’s *t*-test or ANOVA (analysis of variance).
